# Perioperative Rhabdomyolysis in Obese Individuals Undergoing Bariatric Surgery: Current Status

**DOI:** 10.3390/healthcare12202029

**Published:** 2024-10-12

**Authors:** Gilberto Duarte-Medrano, Natalia Nuño-Lámbarri, Marissa Minutti-Palacios, Guillermo Dominguez-Cherit, Analucia Dominguez-Franco, Luigi La Via, Daniele Salvatore Paternò, Massimiliano Sorbello

**Affiliations:** 1 Anesthesiology Department, Medica Sur Clinic & Foundation, Mexico City 14050, Mexico; minuttita@gmail.com (M.M.-P.); g.dominguez.cherit@gmail.com (G.D.-C.); 2 Translational Research Unit, Medica Sur Clinic & Foundation, Mexico City 14050, Mexico; analuciadominguez.f@gmail.com; 3 Surgery Department, Faculty of Medicine, The National Autonomous University of Mexico (UNAM), Mexico City 04510, Mexico; 4 School of Medicine, Tecnológico de Monterrey, Mexico City 14380, Mexico; 5 Department of Anesthesia and Intensive Care, University Hospital Policlinico “G. Rodolico–San Marco”, 95123 Catania, Italy; luigilavia7@gmail.com; 6 UOC Rianimazione, Hospital “Giovanni Paolo II”, 97100 Ragusa, Italy; paternomd@icloud.com (D.S.P.); maxsorbello@gmail.com (M.S.); 7 Anesthesia and Intensive Care, School of Medicine, KORE University, 94100 Enna, Italy

**Keywords:** rhabdomyolysis, anesthesia, bariatric, surgery, complication, obesity

## Abstract

One potential complication in bariatric surgery is rhabdomyolysis, which is a condition involving muscle tissue damage that can significantly impact a patient’s health. The causes of rhabdomyolysis can be broadly classified into two major categories: traumatic and non-traumatic. Early investigations into the development of intraoperative rhabdomyolysis in bariatric surgery identified the main risk factors as tissue compression—primarily affecting the lower extremities, gluteal muscles, and lumbar region—as well as prolonged periods of immobilization. Clinically, rhabdomyolysis is typically suspected when a patient presents with muscle pain, weakness, and potentially dark urine or even anuria. However, the most reliable biomarker for rhabdomyolysis is elevated serum creatine kinase levels. The primary goal in managing hydration is to correct intravascular volume depletion, with solutions such as Lactated Ringer’s or 0.9% saline being appropriate options for resuscitation. Perioperative diagnosis of rhabdomyolysis poses a significant challenge for anesthesiologists, requiring a high degree of clinical suspicion, particularly in bariatric patients. In this vulnerable population, prevention is crucial. The success of treatment depends on its early initiation; however, there are still significant limitations in the therapies available to prevent renal injury secondary to rhabdomyolysis.

## 1. Introduction

Obesity has emerged as one of the most critical global health challenges of the 21st century. Currently, an estimated 1.9 billion people are classified as overweight, with 609 million considered obese, representing a staggering 39% of the global population. This highlights the extensive and pervasive nature of the epidemic [1]. The burden of obesity is unevenly distributed worldwide, with regions such as the Americas and Europe experiencing the highest prevalence rates. Countries like the United States and Mexico consistently rank among those with the highest obesity levels. While historically obesity was more prevalent among women, particularly in the 1980s, recent trends show a more balanced distribution between sexes, with rising obesity rates affecting both men and women [2].

Looking ahead, the projections are alarming. By 2030, nearly 58% of the global population is expected to be either overweight or obese [3]. This anticipated rise underscores the urgent need for effective interventions and preventive strategies to address this growing epidemic. Among the various approaches to managing obesity, bariatric surgery has emerged as one of the most effective and lasting solutions, particularly in cases where pharmacological treatments have failed. As an elective procedure, bariatric surgery offers numerous benefits, including significant and sustained weight loss, improvement or resolution of obesity-related comorbidities, and an overall enhancement in the patient’s quality of life. Currently, the most performed bariatric procedures are sleeve gastrectomy and Roux-en-Y gastric bypass. Notably, about 97% of these surgeries are conducted using laparoscopic techniques, which are associated with fewer complications and quicker recovery times compared to open surgery [4].

The anesthetic management of obese patients undergoing bariatric surgery presents unique and significant challenges, primarily due to the altered pathophysiological characteristics in these individuals, which can complicate the perioperative course. One particularly concerning complication during bariatric surgery is intraoperative rhabdomyolysis. Rhabdomyolysis is a complex, interdisciplinary condition characterized by a breakdown of muscle tissue, which can lead to serious and potentially life-threatening consequences if not promptly recognized and treated [5]. Understanding the pathophysiology of rhabdomyolysis, along with its diagnosis, management, and prognosis in the postoperative period, is crucial for healthcare providers caring for bariatric surgery patients.

This literature review aims to provide a comprehensive analysis of intraoperative rhabdomyolysis in patients undergoing bariatric surgery. By examining current research, the review seeks to clarify the pathophysiological mechanisms underlying rhabdomyolysis, explore the diagnostic challenges, and discuss management strategies as well as prognostic implications for patients in the postoperative period.

## 2. Pathophysiology

The earliest recorded description of rhabdomyolysis dates back to the observations of Antonio D’Antona, who meticulously documented a series of patients suffering from severe muscle injuries following the devastating 1908 earthquake in the Messina region of southern Italy [6]. These injuries, which were caused by violent compression, led to the destruction of muscle tissue—a phenomenon D’Antona recognized as a multifactorial pathology. Although the exact mechanisms of rhabdomyolysis were not well understood at the time, his observations laid the foundation for recognizing this condition as a significant clinical issue with a complex pathophysiology.

**Figure 1:**
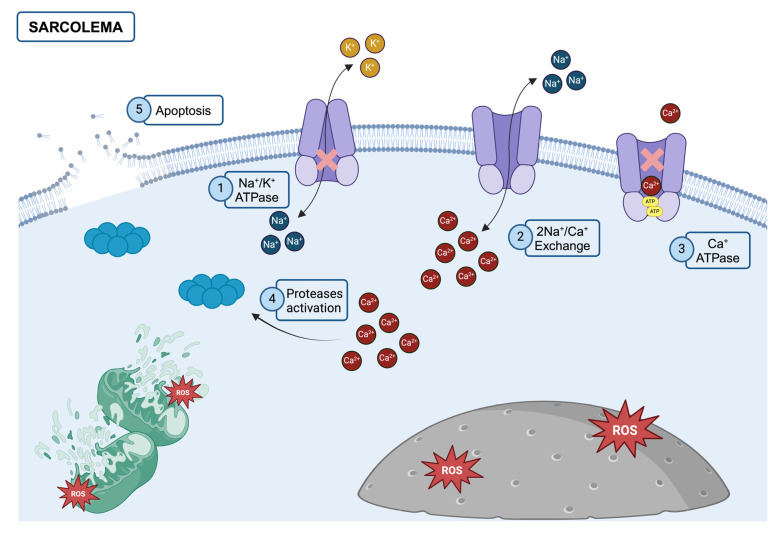
Molecular mechanism of rhabdomyolysis.

The currently accepted theory explains the process of myocyte destruction, which is central to the pathogenesis of rhabdomyolysis. This process begins with a reduction in adenosine triphosphate (ATP), which is the primary energy currency of cells. ATP is crucial for maintaining various cellular functions, including the regulation of ion gradients across cell membranes. When ATP levels decrease, calcium ATPase pumps, which are responsible for keeping intracellular calcium ion (Ca^2+^) concentrations low, become impaired. As these pumps fail due to ATP depletion, pathological accumulation of calcium occurs within myocytes (see Figure 1). Calcium ions play a critical role in muscle contraction by interacting with myofibrils, which are the contractile units within muscle cells. However, in the context of rhabdomyolysis, excessive calcium causes persistent and uncontrolled contraction of myofibrils. This continuous contraction not only exhausts the already depleted energy reserves but also leads to the activation of various proteolytic enzymes, including proteases and phospholipases. These enzymes contribute to the breakdown of cellular components, ultimately resulting in destruction of the myocyte [7].

The death of muscle cells is a critical event in the pathophysiology of rhabdomyolysis. When the integrity of the myocyte membrane is compromised, intracellular contents—such as potassium, phosphate, uric acid, and myoglobin—are released into the bloodstream. This sudden release can have several harmful effects on the body. For example, the rapid influx of potassium can lead to hyperkalemia, which is a potentially life-threatening condition that may cause cardiac arrhythmias. Likewise, hyperuricemia, which is caused by the breakdown of purines in the cell nucleus, can result in kidney damage and contribute to acute renal failure. Furthermore, elevated calcium and phosphate levels in the bloodstream often precipitate and deposit in soft tissues, leading to calcification. This process is exacerbated by the acidic environment generated by anaerobic metabolism, which occurs due to muscle ischemia. An inadequate oxygen supply forces the cells to rely on anaerobic pathways for energy, resulting in the accumulation of lactic acid and a subsequent drop in pH. This acidic environment not only worsens cellular injury but also promotes the deposition of calcium salts in damaged tissues [7].

The pathophysiological cascade described above underscores the multifaceted and complex nature of rhabdomyolysis (Figure 1). This condition arises not only from the direct destruction of muscle tissue but also from a series of biochemical and metabolic disturbances that can have wide-ranging systemic effects. A thorough understanding of these mechanisms is essential for the effective management of rhabdomyolysis, particularly in the context of bariatric surgery, where the risk is elevated due to the unique challenges associated with the surgical and anesthetic care of obese patients.

## 3. Etiology: Non-Traumatic Causes, Metabolic Disorders, and Physical Activity

Among the various causes of rhabdomyolysis, they can be classified into two major categories: traumatic and non-traumatic (Table 1).

Traumatic causes are primarily derived from accidents, conflicts, or natural disasters, as described during the early 19th century, secondary to crushing injuries and prolonged immobilization [8].

### 3.1. Bariatric Surgery as a Risk Factor for the Development of Rhabdomyolysis

Early investigations into the development of intraoperative rhabdomyolysis in bariatric surgery identified key risk factors, including tissue compression, particularly in the lower extremities, gluteal muscles, and lumbar region. Prolonged immobilization during surgery was also recognized as a significant contributor to muscle damage. These risks were more prevalent during the early years of bariatric surgery when surgical techniques were less advanced, leading to extended operating times and a higher risk of complications [9].

**Figure 2:**
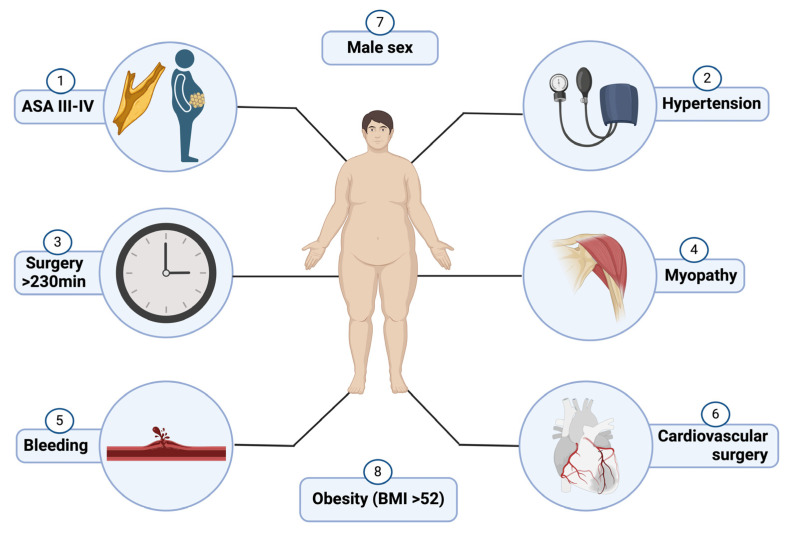
Perioperative risk factors for rhabdomyolysis.

These challenges were particularly pronounced in the early days of bariatric surgery, where extended surgical times often necessitated additional training in these techniques [10]. Early research highlighted the strong correlation between prolonged surgery and the development of rhabdomyolysis. It was frequently observed that longer procedures, often lasting several hours, were associated with a higher incidence of rhabdomyolysis. This was primarily due to patients remaining immobile for extended periods and being subjected to significant pressure in specific body regions. Despite substantial evidence linking prolonged surgical times with rhabdomyolysis, cases have also been reported in surgeries lasting less than 70 min, suggesting that additional factors may contribute to the condition’s pathogenesis [11,12] (Figure 2).

With the refinement of surgical techniques over the past few decades, there has been a significant reduction in the time patients spend in the operating room. However, despite these advancements, bariatric surgery patients remain at risk for developing rhabdomyolysis due to various independent factors inherent to this population.

In a comprehensive review conducted by Chakravartty et al., which included approximately 22 studies covering 145 patients who developed rhabdomyolysis following bariatric procedures, several key risk factors were identified. This analysis revealed that specific patient characteristics and procedural variables were strongly associated with an increased likelihood of developing rhabdomyolysis. Notably, male patients were found to be at higher risk, with a body mass index (BMI) exceeding 52 kg/m^2^ being a significant predictor. The elevated risk in male patients may be attributed to differences in body composition, as males typically have a higher proportion of muscle mass, which may predispose them to greater muscle damage during surgery. Patients who developed rhabdomyolysis were more likely to be male, with a BMI over 52 kg/m^2^ and a surgery duration exceeding 255 min. Among these patients, 14% developed acute kidney injury [13].

### 3.2. Vascular Obstruction Due to Surgery

Vascular obstruction during surgical procedures on both small and large blood vessels is a well-documented contributor to the development of rhabdomyolysis. This is particularly evident in surgeries involving major vascular interventions, where blood flow is intentionally interrupted to facilitate the procedure. A classic example is aortic cross-clamping during abdominal aneurysm surgery. In these cases, the temporary cessation of blood flow to large portions of the body, especially the lower extremities, leads to ischemia, which subsequently causes muscle tissue damage and the release of intracellular components into the bloodstream. This ischemia–reperfusion injury, triggered by the restoration of blood flow after a period of occlusion, is a key factor in the pathogenesis of rhabdomyolysis. Reported incidence rates of rhabdomyolysis following vascular surgeries, such as aortic cross-clamping, vary widely, ranging from 5% to as high as 25% [14,15].

Predisposition to acute renal injury in patients undergoing cardiopulmonary bypass is particularly concerning. The kidneys are responsible for filtering excess myoglobin from the blood, but high levels of this protein, combined with factors like reduced renal perfusion and the use of nephrotoxic agents during surgery, can lead to acute kidney injury. Additionally, in cardiopulmonary bypass procedures, early postoperative increases in myoglobin levels and a heightened risk of acute renal damage have been observed in patients requiring pump support during surgery [16].

### 3.3. Malignant Hyperthermia

Malignant hyperthermia is a rare pharmacogenetic disorder with potentially fatal outcomes if not treated promptly. It results from a hypermetabolic response in muscle cells triggered by exposure to certain anesthetic agents, such as halogenated inhalation anesthetics—most notably halothane—and neuromuscular blockers like succinylcholine. The estimated incidence of malignant hyperthermia ranges from 1 in 10,000 to 1 in 250,000 procedures [17]. The condition is more prevalent in younger populations, and patients with mutations in the RYR1 and CACNA1S genes—both of which are involved in calcium channel regulation—are at higher risk. Classic symptoms of malignant hyperthermia include tachycardia, tachypnea, elevated carbon dioxide production, hyperthermia, metabolic acidosis, and rhabdomyolysis. The treatment of choice is dantrolene, which is a muscle relaxant that works by inhibiting calcium release from the sarcoplasmic reticulum, effectively antagonizing ryanodine receptors and reducing the hypermetabolic response [18].

### 3.4. Drugs

The use of certain drugs contributes to one of the main causes of rhabdomyolysis development, with statins being a notable example, with an annual incidence described in the literature of 3.5–5:100,000 patients. Among antibiotics, rhabdomyolysis has been described with macrolides, fluoroquinolones, and daptomycin from the cyclic lipopeptide family. Several drugs have been associated with the development of rhabdomyolysis. These include commonly used substances such as alcohol, benzodiazepines, and barbiturates. Certain medications used for cholesterol management, like statins and fibrates, have also been implicated. Colchicine, often used for gout, and diuretics are other known contributors. Specific antibiotics, such as daptomycin, and anesthetic agents, including halogenated anesthetics, succinylcholine, and propofol, may increase the risk as well. Additionally, certain antipsychotic medications have been linked to rhabdomyolysis, highlighting the broad range of drugs that can potentially trigger this condition [19,20].

### 3.5. Metabolic Disorders and Physical Activity

Several metabolic disorders, such as refeeding syndrome in malnourished patients, can lead to rhabdomyolysis due to the excessive phosphate consumption required for ATP production [21]. Likewise, extreme body temperatures—whether from induced hypothermia or burns—have been recognized as potential triggers of rhabdomyolysis.

Glycogen storage diseases (GSDs) are inherited metabolic disorders characterized by the abnormal storage or use of glycogen. The most common GSDs associated with rhabdomyolysis include the following: 

McArdle disease: This condition results from a deficiency in myophosphorylase, which is the enzyme responsible for breaking down glycogen in muscle cells. McArdle disease typically presents with exercise intolerance, muscle pain, and early fatigue. During strenuous or prolonged exercise, muscles are unable to access glycogen stores, leading to energy depletion and muscle breakdown, which significantly raises the risk of rhabdomyolysis.

Pompe disease: This condition involves a deficiency in the enzyme acid alpha-glucosidase, which breaks down glycogen within lysosomes. Although more commonly associated with cardiomyopathy and respiratory difficulties, muscle weakness and rhabdomyolysis can occur, particularly in the late-onset form of the disease.

Cori or Forbes disease: This is caused by a deficiency in the glycogen debranching enzyme GSD III and can lead to muscle weakness and an increased risk of rhabdomyolysis, particularly during exercise or fasting when glycogen breakdown is crucial for energy production [22].

Mitochondrial myopathies are disorders of the mitochondrial respiratory chain, which is responsible for ATP production. Impaired mitochondrial function results in energy deficits, particularly during physical activity, increasing susceptibility to muscle damage and rhabdomyolysis. One of the more common mitochondrial disorders associated with recurrent rhabdomyolysis, carnitine palmitoyltransferase II deficiency, affects the transport of long-chain fatty acids into mitochondria for beta-oxidation. Patients with this condition experience episodes of rhabdomyolysis triggered by prolonged exercise, fasting, or cold exposure, when fatty acid oxidation becomes the primary energy source [23].

Physical activity is also a well-documented non-traumatic cause of rhabdomyolysis, particularly after prolonged or intense exercise. Patients at a higher risk often have poor baseline conditioning, engage in physically demanding activities, and experience dehydration, obesity, or substance abuse, such as tobacco consumption. In cases of alcohol-induced rhabdomyolysis, the condition is typically related to muscle ischemia caused by prolonged compression due to the sedative effects of alcohol [24].

## 4. Diagnosis

The classic clinical presentation of rhabdomyolysis is typically characterized by muscle pain, weakness, and possibly dark urine or even anuria. Serum creatine kinase (CK) is the biomarker most strongly associated with rhabdomyolysis, with levels considered suggestive of the condition when they exceed five times the upper limit of normal or surpass 1000 IU/L. It is important to note that CK levels rise progressively during the first 12 h and peak between 3 to 5 days. Additionally, other serum markers, such as lactate dehydrogenase (LDH), potassium, creatinine, aspartate aminotransferase (AST), and myoglobin, are often elevated. Urinalysis can be helpful in detecting myoglobin, especially when serum levels exceed 0.3 mg/L, although its specificity varies widely, ranging from 30% to 80% [25]. Furthermore, increases in prothrombin time and fibrinogen levels may indicate the presence of disseminated intravascular coagulation (DIC). Metabolic acidosis is also a common acid–base disturbance observed in rhabdomyolysis [26].

In a 2017 study conducted by Moulla et al., researchers aimed to establish postoperative serum myoglobin levels associated with clinical factors for predicting complications in patients undergoing bariatric surgery. The study observed 281 patients over a 3-year period. The findings indicated that in patients with a BMI of ≥60 kg/m^2^ and surgical procedures lasting ≥160 min, serum myoglobin levels increased by 32%, with values reaching ≥3000 ng/mL [27].

A tool developed by McMahon et al. provides a method for identifying patients at high risk of renal failure and mortality. This scale (Table 2) assigns scores based on various clinical factors, where a score of ≥6 suggests the need for initiating renal protective therapy, including intensive fluid management. A score of ≥10 is associated with a 52% mortality rate or the requirement for renal replacement therapy [28].

## 5. Complications

Rhabdomyolysis is a rare but potentially life-threatening complication, particularly in patients with significant risk factors such as those undergoing bariatric surgery. The risk of rhabdomyolysis increases with prolonged surgery times, especially when procedures exceed 180 min, involve open Roux-en-Y gastric bypass (RYGB), or when the patient’s BMI is greater than 50 kg/m^2^. In these high-risk cases, early measurement of creatine kinase levels is recommended to detect rhabdomyolysis and prevent its potentially fatal complications [9].

Once rhabdomyolysis is diagnosed, prompt and adequate fluid replacement is critical. Administering fluids at a rate of 200 to 300 mL per hour, with a total daily volume of 10 to 12 L, can help maintain renal function and reduce mortality. Initiating fluid therapy within six hours, followed by appropriate diuresis, is particularly effective in preventing acute renal failure (ARF), which is the most common and severe complication associated with rhabdomyolysis [29,30].

Rhabdomyolysis is a nonspecific clinical syndrome that can lead to severe complications, including electrolyte imbalances, hypovolemia, metabolic acidosis, coagulopathies, and ARF. The mortality rate for patients with rhabdomyolysis complicated by ARF is approximately 20%. Other complications include hepatic injury, hyperkalemia, disseminated intravascular coagulation, thrombosis, and hemorrhage. Among these, ARF is closely linked to elevated potassium levels and is often exacerbated by hypovolemia and metabolic acidosis. Although the exact mechanism of ARF in rhabdomyolysis is not fully understood, factors such as renal vasoconstriction due to hypovolemia and cytokine cascade activation by hemoproteins are thought to play a significant role. This underscores the importance of aggressive fluid therapy to prevent ARF and other life-threatening outcomes [30].

## 6. Perioperative Recommendations

**Figure 3:**
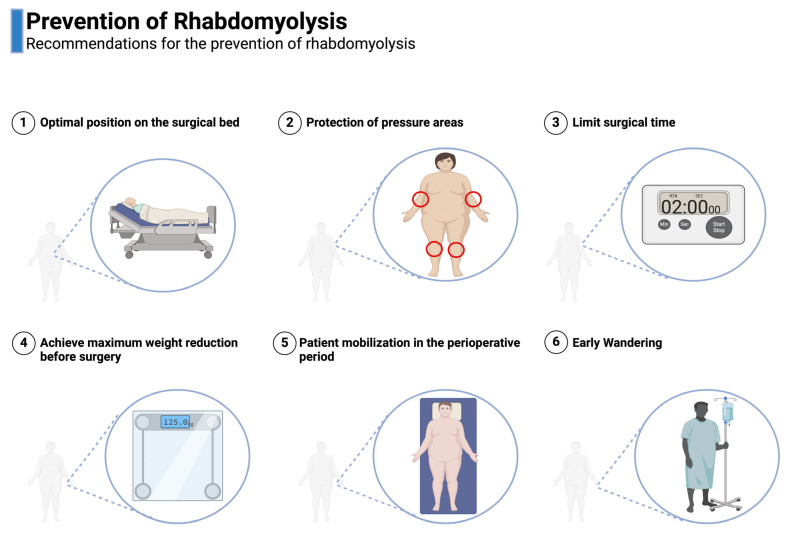
Prevention of rhabdomyolysis.

Perioperative management for patients at risk of rhabdomyolysis, particularly in bariatric surgery, is crucial. Effective strategies include implementing preventive measures such as weight loss [31]. During surgery, it is essential to prioritize patient positioning, utilize pneumatic beds, and apply compression bandaging, while also aiming to limit the duration of the surgery to under 160 min [5,9,10]. Figure 3 illustrates the preventive measures for rhabdomyolysis in bariatric surgery.

## 7. Treatment

The primary objective of hydration management is to correct intravascular volume depletion. Solutions such as Lactated Ringer’s or 0.9% saline solution are commonly used for resuscitation. Some authors recommend initiating treatment with 1 L boluses, followed by titration to achieve a urine output of 200–300 mL/h or 3–4 mL/kg/h [5,32]. Conversely, the American Association for Surgery of Trauma and Critical Care advises starting rehydration at 400 mL/h, aiming for a urine output of 1–3 mL/kg/h. Currently, the use of bicarbonate or ASA diuretics has not been shown to effectively prevent acute kidney injury secondary to rhabdomyolysis, and their use is, therefore, not recommended [7,33,34].

When considering renal replacement therapy for patients who develop acute kidney injury secondary to rhabdomyolysis, the decision should be guided by the extent of renal damage and the patient’s overall clinical condition [23,35]. However, its effectiveness in preventing acute kidney injury remains unestablished [36,37].

## 8. Future Perspectives

The rising prevalence of obesity worldwide underscores the need for continued research and advancements in bariatric surgery and the management of associated complications such as rhabdomyolysis. As surgical techniques advance, it is anticipated that the incidence of intraoperative rhabdomyolysis will decrease. However, the increasing number of bariatric procedures globally necessitates ongoing efforts to identify and mitigate risk factors. Future research should focus on developing more precise diagnostic tools and biomarkers for the early detection of rhabdomyolysis, enabling the timely intervention and prevention of severe complications. Additionally, investigating genetic factors that contribute to individual susceptibility to rhabdomyolysis could lead to personalized risk assessments and tailored preventive strategies. 

Further investigation is also needed in the perioperative management of bariatric patients at risk of rhabdomyolysis. Continuously assessing and refining practices such as patient positioning, limiting surgical duration, and implementing preventive measures like pneumatic beds and compression bandaging will be essential in minimizing this complication. As our understanding of the pathophysiology of rhabdomyolysis improves, novel therapeutic approaches may emerge. While current treatment focuses primarily on supportive care and the prevention of acute kidney injury, targeted therapies addressing the underlying mechanisms of muscle damage could potentially enhance patient outcomes.

Finally, the multidisciplinary nature of managing rhabdomyolysis in bariatric surgery highlights the importance of collaboration among healthcare professionals, including surgeons, anesthesiologists, and critical care specialists. Establishing standardized protocols and guidelines for the prevention, diagnosis, and management of this complication will be crucial for optimizing patient care and outcomes in the future

## 9. Conclusions

Rhabdomyolysis, while rare in the general surgical population, poses a substantial risk to patients, particularly those with major risk factors such as individuals undergoing bariatric surgery. For anesthesiologists, diagnosing rhabdomyolysis perioperatively presents a significant challenge, necessitating a high level of clinical suspicion. In this vulnerable population, prevention is crucial and forms the cornerstone of effective management. Early intervention is key to successful treatment. However, current therapies for preventing renal injury secondary to rhabdomyolysis have notable limitations, and existing evidence does not support their widespread use.

## Figures and Tables

**Table 1:** Etiology of rhabdomyolysis.

Traumatic	Not Traumatic
Electrocution	Xenobiotics (drugs, toxins)
Crushing accidents	Hydroelectrolytic alterations
Compartment syndrome	Exercise
	Surgery
	Infections
	Genetic alterations

**Table 2:** McMahon scale.

Variable	Score
Age (years)	
>50 a <70	1.5
>70 a <80	2.5
>80	3
Woman	1
Initial creatinine (mg/dL)	
1.4–2.2	1.5
>2.2	3
Initial calcium < 7.5 mg/dl	2
Initial CPK > 40,000 U/L	2
Initial phosphate (mg/dL)	
4.0–5.4	1.5
>5.4	3
Initial bicarbonate < 19 mEq/L	2
Etiology not derived from exercise, statins, myositis, syncope, or seizures	3

## Data Availability

Data sharing is not applicable; no new data were created or analyzed in this study.
